# The precise magic of CRISPR

**DOI:** 10.1002/2211-5463.13195

**Published:** 2021-06-01

**Authors:** Alexander Kondrashov

**Affiliations:** ^1^ Division of Cancer and Stem Cells University of Nottingham Biodiscovery Institute UK; ^2^ Centre of Membrane Proteins and Receptors (COMPARE) Universities of Birmingham and Nottingham Midlands UK

**Keywords:** cancer, CRISPR/Cas9, genome editing, polyploid cells

## Abstract

In this issue of *FEBS Open Bio*, Shen Li *et al.*, in the laboratory of Hector L. Franco (University of North Carolina), provide a proof‐of‐principle solution for correcting all copies of a gene in the widely used MCF7 breast cancer cell line. The gene for the FOXA1 pioneer transcription factor is localised on chromosome 14, which is present at least 4–5 times in MCF7 cells. To achieve their goal, the authors used a ‘classical’ version of the CRISPR/Cas9 system. Both sgRNA and Cas9 components were expressed from a single vector, which also has a puromycin resistance cassette; this is an essential module for the chosen strategy, because it ensures expression of both sgRNA and Cas9 in selected cells. A targeting template in the form of nonlinearised plasmid was shown to have the best efficiency and was used to introduce a substitution at position 295 in the gene encoding FOXA1 to change a codon encoding lysine into a codon encoding glutamine (K295Q). The strategy suggested by Li and co‐authors is an important development towards genome editing of multiple copy genes in a polyploid environment like cancer cells. One important application of the technique could be in creating models to study the role of single nucleotide polymorphisms in cancer progression and metastasis. Isogenic cancer lines carrying polymorphic variants of key drug targets could be used to optimise anticancer treatment protocols, laying a foundation for personalised therapy.

## Graphical abstract

This *Insight* article introduces the work of Shen Li *et al*., who provide a proof‐of‐principle solution for correcting all copies of a gene in the widely used MCF7 breast cancer cell line. One important application of the technique could be in creating models to study the role of single nucleotide polymorphisms in cancer progression and metastasis.

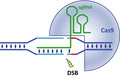

**Abbreviations**
DSB: double‐strand break
HDR: homology directed repair
PAM: protospacer adjacent motif

Gene editing refers to a group of methods that allow the introduction of precise changes in the DNA of a cell. Most of these methods require a double‐strand break (DSB) in the DNA near the desired change and a template DNA that spans the break site and includes the intended modification [1]. The latter is used by a DNA repair pathway called homology directed repair (HDR), which uses the template to patch the break and at the same time introduces the desired modification into the gene. Earlier versions of this technology relied on the presence of endogenous random DNA breaks and therefore were very inefficient and time‐consuming [2]. The introduction of programmable nucleases, enzymes that can be designed to recognise and cleave DNA sequences at specific genome locations, significantly improved our ability to manipulate genomes [3].

The CRISPR‐based programmable nucleases are relative newcomers on the scene, but in a short time they have conquered the field due to their simplicity in design and their cost‐effectiveness in comparison with zinc‐finger or TALEN‐based platforms [4, 5, 6]. One popular version of the system is derived from the bacterium *Streptococcus pyogenes* and consists of two essential modules: single guide RNA (sgRNA or simply gRNA) and Cas9 protein [6, 7, 8].

Cas9 and guide RNA form a complex, which can bind to DNA and cleave it, generating a DSB. The specificity of target DNA recognition is determined by 20 bases positioned at the start of the sgRNA, which are complementary to the so‐called protospacer, which are 20 bases that have been selected in the targeted DNA region (Fig. 1). The other important determinant for successful cleavage at the right site is the protospacer adjacent motif (PAM) [6]. The latter is positioned immediately downstream of the protospacer and consists of three nucleotides, of which the last two have to be the nucleobase guanine (or G). Therefore, in order for sgRNA/Cas9 nuclease to target a desired location in the genome, one only needs to find a GG doublet in either DNA strand near the target location and design a sgRNA able to recognise the 20 nucleotides upstream of it (Fig. 1). It is important to keep in mind that similar sequences could exist in other parts of the genome. These sequences, called off‐targets, can be recognised and cleaved by sgRNA/Cas9 complex too, leading to the accumulation of unintended modifications.

**Fig. 1:**
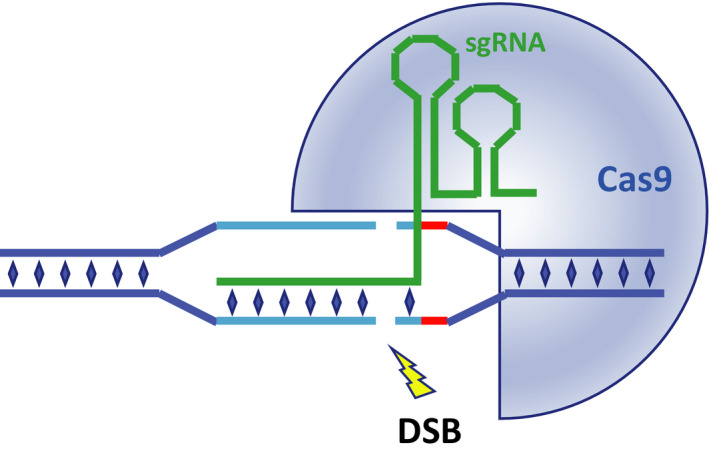
Interaction of sgRNA/Cas9 complex with target DNA. The specificity of the interaction between sgRNA/Cas9 complex and genomic DNA is determined by complementarity between the terminal 20 nucleotides of sgRNA (green) and the opposite strand of protospacer motif (light blue) within genomic DNA. The protospacer adjacent motif (PAM, red in the diagram) is another essential requirement. It should contain an NGG triplet, which is identified by the *S. pyogenes* CRISPR/Cas9 system. The binding of sgRNA/Cas9 to target DNA induces activation of Cas9 (shown as a blue ¾ circle) nuclease activity, leading to generation of a double‐strand break (DSB).

Not all cell types can be readily subjected to HDR‐mediated gene editing due to their differential repair and proliferative capacities [9]. For example, primary cells are restricted in their proliferation potential, making it hard to obtain sufficient genetically identical material from a single edited cell. While some cultured cancer lines can be a good platform for precise gene manipulations, others are almost impossible to modify due to defects in their DNA repair machinery. Moreover, many cancer cell lines contain multiple copies of chromosomes due to genome instability; this makes genome editing a very challenging process, as more than two chromosomes may have to be modified to change all copies of a gene. In order to successfully perform gene editing in these model systems one should apply a specific strategy for each case. It is also should be noted, that in higher eukaryotes, the HDR pathway is not the predominant choice for DSB repair [10]; this fact introduces an additional level of complexity when precise genome editing is required.

In this issue of *FEBS Open Bio*, Shen Li *et al*., in the laboratory of Hector L. Franco (University of North Carolina), provide a proof‐of‐principle solution for correcting all copies of a gene in the widely used MCF7 breast cancer cell line [11]. The gene for the FOXA1 pioneer transcription factor is localised on chromosome 14, which is present at least 4–5 times in MCF7 cells. To achieve their goal, the authors used a ‘classical’ version of the CRISPR/Cas9 system. Both sgRNA and Cas9 components were expressed from a single vector, which also has a puromycin resistance cassette [12]; this is an essential module for the chosen strategy, because it ensures expression of both sgRNA and Cas9 in selected cells. A targeting template in the form of nonlinearised plasmid was shown to have the best efficiency and was used to introduce a substitution at position 295 in the gene encoding FOXA1 to change a codon encoding lysine into a codon encoding glutamine (K295Q). The resulting modification of the FOXA1 protein, known to be able to displace histone H1, is of special interest as it could potentially create a more relaxed state of chromatin – a complex of DNA and specific proteins (histones) able to pack DNA into regular compact fibres [13]. The authors considered that not all copies are likely to be repaired simultaneously. Some copies might remain intact or contain undesired insertion and deletion mutations (indels), which can be introduced by other types of DNA repair where DSBs occur. Therefore, they selected additional sgRNAs in adjacent regions and introduced silent mutations in the repair template, which should prevent repeated cleavage after the first modification has taken place.

Using various techniques, the authors confirmed successful editing of this and several other positions in independent experiments. The range of achieved efficiencies was between 28% and 100% per cent after only a single round of transfection. Interestingly, all alleles contained either correctly introduced modifications or contained indels, which means that the strategy used by the authors allows 100% targeting (successful cleavage) by the Cas9/gRNA complex. This result is due to the selection procedure, which secures the presence of active nuclease in the timeframe of editing. The use of a selection marker, which is present on the Cas9/gRNA expression vector rather than on the template vector, assures ‘clean’ single step editing because it does not require an additional cassette removal step, unlike other gene editing strategies, which use a selection marker within the targeting vector itself [14] However, a potential adverse effect of the approach chosen by Li and co‐authors could be random integration of gRNA/Cas9 expression vector in undesirable genome locations. Several other aspects of the strategy could be optimised in the future, such as the lifetime of the active nuclease within the cell, as a longer presence will increase the probability of off‐target activity. In addition, the number of silent modifications needed to prevent repeated cleavage by the gRNA/Cas9 complex should be kept to a minimum. These modifications might affect codon usage bias, the phenomenon of unequal representation of synonymous codons within genes or genomes, and influence the level of expression of not only the targeted gene, but other genes as well, especially when the gene is highly expressed [15].

The strategy suggested by Li and co‐authors is an important development towards genome editing of multiple copy genes in a polyploid environment like cancer cells. One important application of the technique could be in creating models to study the role of single nucleotide polymorphisms in cancer progression and metastasis [16, 17]. Isogenic cancer lines carrying polymorphic variants of key drug targets could be used to optimise anticancer treatment protocols, laying a foundation for personalised therapy.

## Conflict of interest

The authors declare no conflict of interest.

## Author contribution

AK wrote the article and prepared the figure.
